# A Framework for Supervising Lifestyle Diseases Using Long-Term Activity Monitoring

**DOI:** 10.3390/s120505363

**Published:** 2012-04-26

**Authors:** Yongkoo Han, Manhyung Han, Sungyoung Lee, A. M. Jehad Sarkar, Young-Koo Lee

**Affiliations:** 1 Department of Computer Engineering, Kyung Hee University, Seocheon-dong, Giheung-gu, Yongin-si, Gyeonggi-do, 446-701, Korea; E-Mails: ykhan@khu.ac.kr (Y.H.); smiley@oslab.khu.ac.kr (M.H.); sylee@oslab.khu.ac.kr (S.L.); 2 Deptartment of Digital Information Engineering, Hankuk University of Foreign Studies, 89 Wangsan-ri, Mohyeon-myeon, Cheoin-gu, 449-791, Korea; E-Mail: jehad@hufs.ac.kr

**Keywords:** sensor system, ubiquitous healthcare system, activity recognition, lifestyle disease, framework

## Abstract

Activity monitoring of a person for a long-term would be helpful for controlling lifestyle associated diseases. Such diseases are often linked with the way a person lives. An unhealthy and irregular standard of living influences the risk of such diseases in the later part of one's life. The symptoms and the initial signs of these diseases are common to the people with irregular lifestyle. In this paper, we propose a novel healthcare framework to manage lifestyle diseases using long-term activity monitoring. The framework recognizes the user's activities with the help of the sensed data in runtime and reports the irregular and unhealthy activity patterns to a doctor and a caregiver. The proposed framework is a hierarchical structure that consists of three modules: activity recognition, activity pattern generation and lifestyle disease prediction. We show that it is possible to assess the possibility of lifestyle diseases from the sensor data. We also show the viability of the proposed framework.

## 1. Introduction

Advancements in sensor technologies give us the opportunity to recognize activities of daily living (ADLs) [1] for a long-period of time. Long-term activity monitoring could be helpful for the caregivers or doctors to monitor user's unhealthy and irregular activity patterns. This not only can reduce of the cost of healthcare but also can avoid unwanted consequences.

Lifestyle diseases such as Alzheimer's disease, atherosclerosis, asthma, cancer, chronic liver disease or cirrhosis, chronic obstructive pulmonary disease, type 2 diabetes, heart disease, metabolic syndrome, nephritis or chronic renal failure, osteoporosis, acne, stroke, depression and obesity, appear to become ever more widespread as countries become more industrialized. These are now one of the main focuses of the researchers due to their high mortality rate [2]. Researchers from all over the world are working for early detection and prevention of such diseases.

A lifestyle disease is associated with the manner a person lives [3]. Lifestyle diseases are different from other diseases because these are potentially preventable, and can be lowered with changes in diet, lifestyle and environment. In particular, an unhealthy and irregular life pattern may increase the risk of lifestyle diseases in the later part of life [3,4].

Monitoring a person's lifestyle over a long period of time can be helpful for the following purposes:

- **Early identification of the lifestyle:** A person is generally at risk of a lifestyle disease when he/she lives his/her life in a certain style. Moreover, initial signs and symptoms of such diseases appear in people with irregular life patterns [5,6]. By long term activity monitoring, it would be possible to identify such styles (e.g., irregular and unhealthy) and determine the risk of the disease before the actual disease appears.
- **Prevention of lifestyle diseases:** It would be possible to prevent a disease at the first place with the identification of the lifestyle. A healthy lifestyle and physical activity help prevent obesity, heart disease, hypertension, diabetes, colon cancer, and premature mortality [7]. A person can assess his/her lifestyle and become aware of any irregular patterns though monitoring daily activity patterns.
- **Management of lifestyle diseases:** A person who has a lifestyle disease should make changes to assure a healthy lifestyle. For example, there is no cure for type 2 diabetes, but it can be effectively controlled by maintaining normal blood-glucose levels. This can be achieved through weight control by following a nutritious and balanced diet, along with regular and stringent exercise [8].

In this paper, we propose a framework for supervising lifestyle diseases with the help of long-term activity monitoring. The framework can produce a significant amount of information to assist the medical practitioners in not only diagnosing a lifestyle disease but also to prevent it. The key idea of the framework is that we monitor target activities that reflect the initial signs and symptoms of lifestyle disease. Since lifestyle diseases appear in people with irregular life patterns, we can predict the risk of the diseases by monitoring the target activities for a long-period of time.

The framework has a hierarchical structure that consists of four layers: (1) activity classification layer, (2) activity pattern generation layer, (3) disease inferring layer and (4) application layer. In the activity classification, target activities are recognized using an activity recognition technique. In the activity pattern generation layer, a regular pattern of each target activity is generated by adopting a statistical modeling approach. Finally, in the disease inferring layer, the risk of lifestyle disease is measured based on the similarity between the daily activity pattern and a predefined disease symptom pattern. In the web-based application layer, the user's current activity, activity pattern, and risk of disease are reported to medical practitioners.

The contributions of this paper are summarized as follows: We propose a healthcare framework for supervising the possibility of lifestyle diseases using long-term activity patterns. These patterns are generated by monitoring ADLs. The current version of the framework is applicable to (but not limited to) a home environment. We consider a set of sensors that are embedded with the daily-life objects (of the environment) such that it is possible to determine the state of an object when it is used. We performed two experiments to validate the performance of the framework. Apart from supervising the lifestyle diseases, the proposed framework has lot of other applications such as elderly monitoring, ADLs pattern analysis and regular lifestyle management. To the best of our knowledge this is the first framework for activity based lifestyle disease prediction.

The remainder of this paper is organized as follows: in the next section, we discuss related works and the study background. In the third section, we explain the details of the proposed healthcare framework. We discuss the experimental results in Section 4 and, finally, present our conclusions and describe our future work in the final section.

## 2. Background and Related Works

Long-term activity monitoring can be regarded as observing human activities on a daily basis for a long period of time. It has lots of applications, especially in healthcare. Based on the application, the long-term activity monitoring systems can be categorized into two types [9]: disease management and health monitoring systems.

Disease management systems use the physiological and psychological data of real-life for managing chronic disorders or health problems, diabetes [10] and obesity [11,12]. Such systems require the user to measure his/her health status (such as blood sugar level or weight) by his/herself and respond to any feedback received. Although such a system can provide good quality service, it could not be general-purpose. It would be difficult for many users to provide self-reported data.

Health monitoring systems [13–17] on the other hand automatically monitor and report the user's health and daily life patterns. For this purpose, such a system uses embedded sensors and/or wearable sensors for accumulating the user's information. Modern day home healthcare systems are mostly health monitoring systems.

Activity-based lifestyle supervising is a type of a health monitoring system. In such systems, lifestyle patterns are measured with the help of statistical data of daily living. In [13,14], Virone *et al.* installed infrared (IR) motion sensors in houses for collecting users' movement data. He then estimated circadian activity rhythm based on the average amount of time a resident spent in each room, and also on the activity level given by the average number of motion events per room. Large deviations from this average time or number are considered as abnormal patterns. The system proposed by Ohta *et al.* in [15] is based on a similar concept with [14], except that the average time spent in each room is estimated for each day. They also included the average movements of a user for detecting any abnormalities.

In [16], Cardinaux *et al.* collected user activity data by installing bed and chair occupancy sensors, passive infra-red movement detectors door contact monitors, and electrical usage sensors in a house. He recognized two activities (sleeping and watching TV) based on a rule-based algorithm. For example, a sleeping period is detected when a bed sensor is fired followed by a sensor “out” event. Then, he modeled normal activity pattern with Gaussian mixture model and detect abnormal activity patterns for each activity. Shin *et al.* [17] extracted three different feature values [activity level, mobility level, and non-response interval (NRI)] by using IR sensors. The activity level represents how many times subject's motion was captured. The mobility level represents subject's movement. The NRI represents a time between the subject's motions. The support vector data description (SVDD) method was used to classify normal behavior patterns and to detect abnormal behavioral patterns based on the three features. Since they consider three features, they can detect more detail abnormal behavioral patterns such as weakness, seizures, falls and severe pain, *etc*.

The above systems deal only with the abnormalities of one or more activities. These systems do not have the ability to determine what or how the abnormalities could affect him/her in the later part of the life. In other words, these systems monitor the user activities for short term and detect any anomalies.

In this paper, we propose a healthcare framework for supervising lifestyle diseases using long-term activity monitoring. We show how long-term activity monitoring technique can be applied for managing lifestyle diseases. Moreover, we adopt activity recognition technique for modeling user activity pattern with various activities such as eating, sleeping, showering, *etc.* The framework does not require any self-reported biomedical data from the user. It is therefore applicable in almost all environments. To the best of our knowledge this is the first approach to monitor lifestyle diseases with the help of long-term activity monitoring.

## 3. Home Healthcare Monitoring Framework

In this section, we explain the proposed framework in detail and show how the risk of lifestyle disease is inferred from the sensor data. The main idea behind the disease inference is to observe the irregularities of the user's daily activities. There could be different types of irregularities of the activity patterns, for example, the frequency of doing an activity is more or less than the usual, the means of doing an activity is not appropriate, or there exists a mismatch of the sequence of activities. For this version of the framework, we have used the frequency of an activity as the primary source for disease inference.

**Figure 1.**
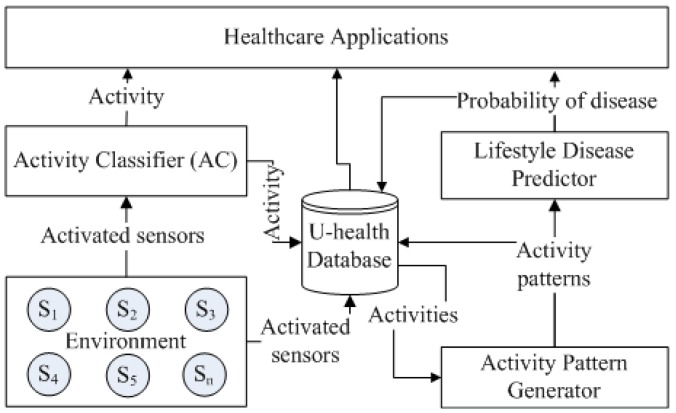
Overall architecture of the proposed healthcare framework.

The overall architecture of the proposed framework is illustrated in Figure 1. It consists of three hierarchically connected modules: activity classification, activity pattern generation and lifestyle disease prediction. The activity classification module is used to recognize the user activity in real-time and provides its output to the activity pattern generation module. The activity pattern generation then determines various parameters such as, daily activity frequency, regular activity frequency, graded activity frequency and daily activity pattern and provides output to the lifestyle disease prediction module. The lifestyle disease prediction module then determines the risk of a lifestyle disease.

The results of the lower level modules are the inputs to the higher level modules. The output of each module are stored in the activity database for providing various healthcare information such as current/last activity, activity frequency and duration, activity pattern, and disease probability. The framework also provides a web-based healthcare application for the clinician and caregiver to monitor the activity database remotely.

### 3.1. Environment

The current version of the framework is applicable to a home environment, however not limited to. The arrangements we need for the framework are follows: a set of lifestyle diseases, the set of disease related activities to monitor, and a set of sensors which are embedded with the home appliances in a way such that it is possible to determine the state of an appliance when it is used.

A set of activities are chosen based on the type of lifestyle diseases we would like to manage. We define such activities as:

**Definition 1. Disease Influenced Activity (DIA):** This is an activity that is influenced by the initial signs and symptoms of a specific disease. It is denoted by *DIA*(*d*) = {*a*_*d*_1__,*a*_*d*_2__,…,*a*_*d*_*n*__} for disease *d* with *n* kinds of DIA.

A set of DIAs per lifestyle disease are shown in Table 1. For example, depression is a serious medical condition that not only affects the mood of a person but also the daily life activities [4]. It has DIAs such as, activity in mild illumination, less sleeping and less talking.

After selecting the activities, the next thing we need is to deploy the sensors to the environment. The sensors are considered to be embedded with the set of home appliances (or objects) which are related to the DIAs. We choose to use binary sensors [16–18], since they have many advantages over the other types of sensors, such as, providing privacy and being inexpensive [18,19].

**Figure 2.**
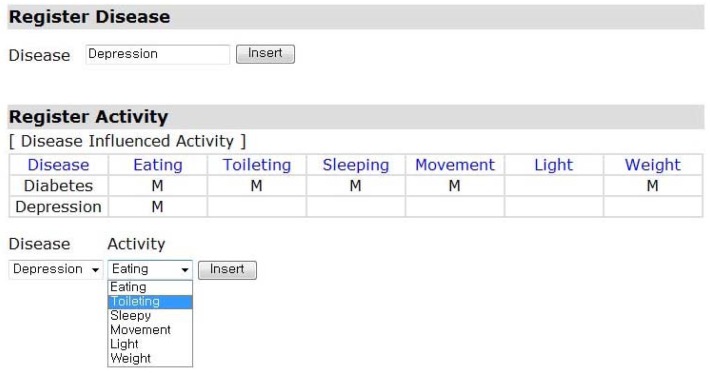
Registration of Disease and DIA.

Figure 2 shows an interface of the disease and DIA registration in the healthcare framework. A disease is registered first, DIAs for the registered disease then are selected for monitoring. For example, the DIAs, eating, toileting, sleeping, movement, and weight are selected for the diabetes. Since the DIA, “activity in mild illumination”, is not the initial sign and symptom for the diabetes, “light” is not selected.

### 3.2. Activity Classifier

The activity classifier is an important component of the system. The accuracy of the disease prediction largely depends on the accuracy of this component.

A set of activity classifiers [18–30] have already been proposed. We choose to use one of these instead of developing our own. Although it is possible to use any type of activity classifiers, we have decided to use embedded sensors-based activity classifier due to its capability of providing privacy, security and inexpensiveness [19–22,29,30]. For the version of the framework, we have employed a C4.5 decision tree based activity classifier because of its high popularity [18,23–25].

**Figure 3.**
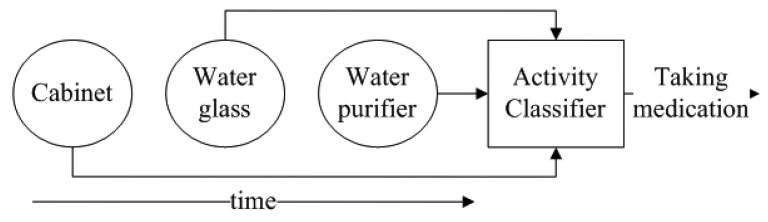
Activity recognition example.

The goal of the activity classifier is to recognize a user's activity, L, depending on the set of objects (embedded with sensors), *s_1_*, *s_2_*, *…*, *s_m_*, he/she used for a given period of time, *T*. For example, let us consider a scenario in which a user is doing an activity by using the following three objects (with embedded sensors), “Cabinet”, “Water glass” and “Purifier” within the last 2 min. The goal of the classifier is to recognize the user's activity (in this case “Taking medication”). Figure 3 shows the scenario of how the classifier classifies the activity.

**Figure 4.**
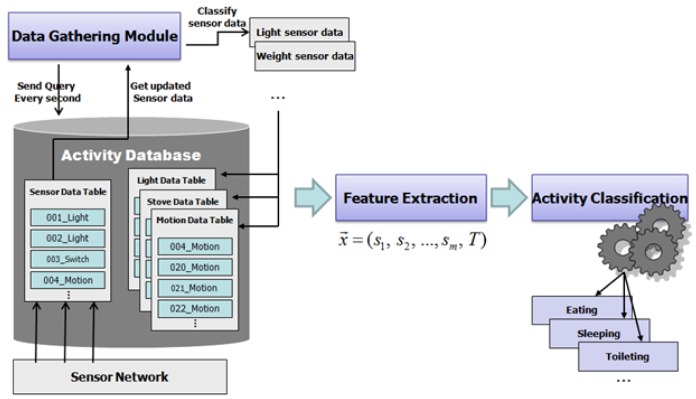
Processing steps of activity classification.

Figure 4 shows the processing steps of the activity classification module. The data gathering module collects the activated sensor (a sensor activates if the object in which it is embedded is used) data for a given time. The sensor data is parsed and stored into the related tables in the activity database. The feature extraction module generates features using the stored sensor data in every predefined time window (e.g., 2 min). The activity classifier classifies the corresponding activity using these features.

**Figure 5.**
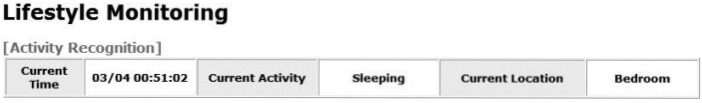
Activity recognition results.

Figure 5 shows the activity recognition result in the web-based healthcare application. The recognized activity and location are displayed in real-time and also storied in the activity database. The activity recognition allows families to take care of their elderly who lives alone. For example, a family member can check whether a father sleeps well or stays too long in the same place.

### 3.3. Activity Pattern Generation

The purpose of this module is to generate the activity pattern for each day. The initial signs and symptoms are represented as the frequencies of DIAs such as frequent drinking in Table 1. Therefore, the activity pattern is modeled by the statistical activity frequency in our work. Before describing more details, we define the following terms related to the activity pattern modeling:

**Definition 2. Daily Activity Frequency (DAF):** is defined as a DIA in a day. This is denoted as *DAF_d_* (*a_i_*), for a DIA, *a_i_*, on day, *d*. For example, if a certain user takes three meals on day 7, then the DAF of that day is denoted as, *DAF*_7_ (*eating*), whose value is 3.

**Figure 6.**
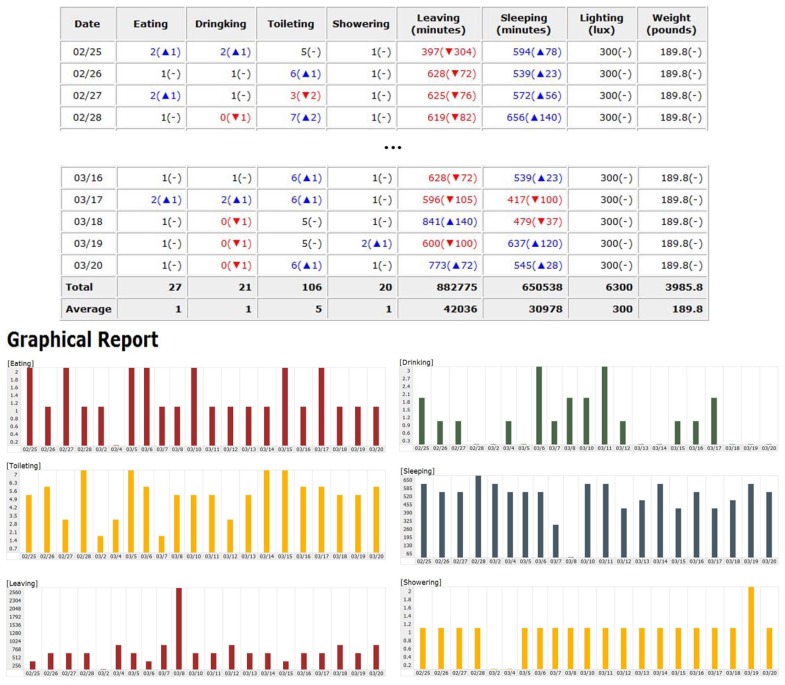
Text- and graphics-based the activity monitoring.

A DAF is determined during the training period. Figure 6 shows the text- and graphics-based DAFs for the subject's dataset of an activity recognition study [22]. DAF for each activity is calculated by aggregating the recognized activity in activity DB once a day. Each entry in the table represents DAF of each DIA. The symbols, ▲ and ▼ represent the increased and decreased daily activity frequency (DAF) respectively, compared to an average DAF. For example, “2(▲ 1)” represents the DAF has increased by 1 for a day. From these results, a doctor or a family member can monitor the subject's daily activity frequency. For example, using this web application, a doctor can monitor an increase or a decrease in the frequency of food consumption per day.

**Definition 3. Regular Activity Frequency (RAF):** is defined as the mean value of a DAF in the training dataset. This is denoted as, *RAF*(*a_i_*) of an activity, *a_i_* and calculated as:
  (1) $$\text{RAF}(a_{i})=\sum_{d=1}^{D}\frac{DAF_{d}(a_{i})}{D}$$

where, *D* is the total days of training. For example, if a user performs “Cooking” for 20 times during the training period that lasts for 25 days, then the *RAF*(*cooking*) will be 0.8.

**Definition 4. Graded Activity Frequency (GAF):** is defined as a deviation of the DAF from RAF. It is denoted as, *GAF_d_*(*a_i_*), for a DIA, *a_i_*, on day, *d*. The GAF has an integer value ranging from −2 to 2 depending on the degree of the deviation from RAF.
**Definition 5. Daily Activity Pattern (DAP)** is defined as the set of all GAFs in a day. It is denoted as, *DAP_d_* = (*GAF_d_* (*a*_1_), *GAF_d_* (*a*_2_),…,*GAF_d_* (*a_n_*)) where *GAF_d_* (*a*_i_) is the GAF in the d^th^ day of a DIA, *a*_i_, and *n* is the total number of DIAs considered.

**Figure 7.**
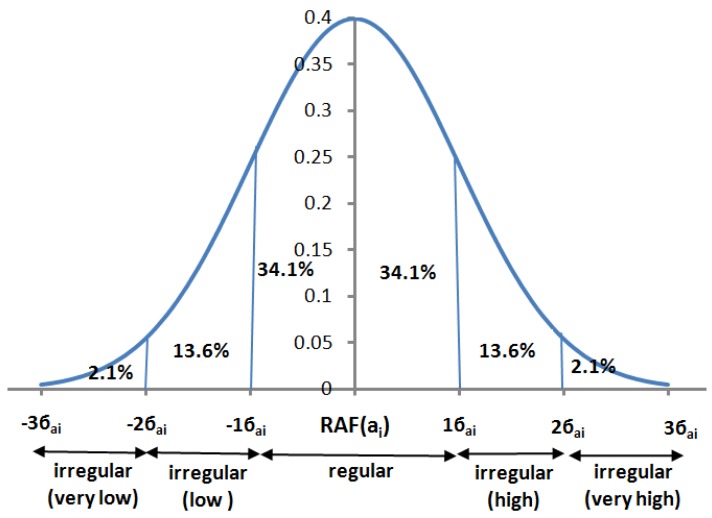
An example of a normal distribution of DAF.

This module generates the DAP each day. For this purpose it first determines the regularities and irregularities of the GAF. We call a GAF to be regular if the standard deviation of a DAF from RAF is 1 (*i.e.*, −1≤ σ ≤ 1). It is to be noted here that we assume that the DAF follows a normal distribution (or bell curve). Figure 7 shows an example of normal distribution of a DAF. We grade each of the activities into 5 bands (as shown in the Figure 5): “very low”, “low”, “regular”, “high” and “very high”.

### 3.4. Lifestyle Disease Prediction

The role of this module is to infer the risk probability of a lifestyle disease per day. It determines the probability of a lifestyle disease from the given disease pattern, *DAP_disease_*, and the performed pattern, *DAP_d_*, of a DIA. *DAP_d_* is determined in the activity pattern generation module, while *DAP_disease_* is determined from the disease symptoms. A doctor decides the disease pattern based on his/her medical knowledge. For example, if diabetes has five kinds of initial signs and symptoms from Table 1, such as frequent liquid intake, frequent eating, frequent sleeping, frequent toileting and low weight, the disease pattern of diabetes could be set as *DAP_diabetes_* = (very high, very high, very high, very high, very low) or (2, 2, 2, 2, −2). The probability of a lifestyle disease, *R_disease_*(*DAP_disease_,DAP_d_*), is measured by the similarity between the *DAP_d_* and the *DAP_disease_* on the d*^th^* day. For this purpose we adopt the Euclidean distance between *DAP_disease_* and *DAP_d_* in Equation (2):

(2) Rdisease(DAPdisease'DAPd)=1−(similarity(DAPdisease'DAPd)MAXDistance)=1−∑i=1n(GAFdisease(ai)−GAFd(ai))2(GAFMAX−GAFMIN)2×n

where, *MAX_distance_* is the maximum distance between *DAP_disease_* and, *DAP_d_, GAF_MAX_* and *GAF_MIN_* are the maximum and the minimum values of the GAF, respectively, and *n* is the total number of given DIAs of the disease. The lifestyle disease prediction module determines the probability of a disease each day and sends the report to the caregiver or doctor if the probability exceeds a certain threshold provided by the doctor.

## 4. Experiments

The objective is to validate the performance of the framework. We have performed two experiments to see how the proposed framework would work for predicting lifestyle diseases using long-term activity monitoring. First, we evaluate the performance of the activity recognition algorithm. Second, we evaluate how the framework would work for disease prediction.

### 4.1. Experiment Setup

In order to evaluate the performance of the framework, the ideal case scenario would be to use a real-world activity dataset which has associated lifestyle diseases. However, to the best of our knowledge there exist no such datasets that particularly show such association. Therefore, we have presumed the relationship between an activity and a lifestyle disease from activity datasets.

**Figure 8.**
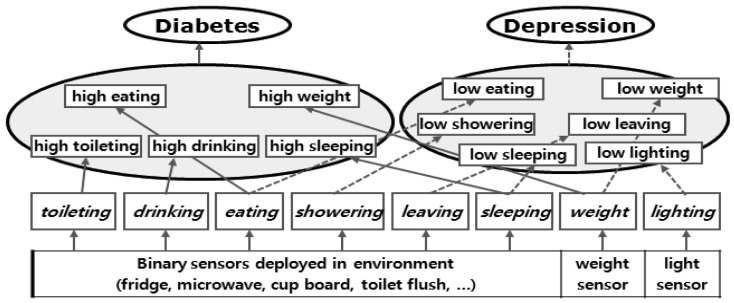
Symptoms of lifestyle disease inference model.

In this paper, we have chosen two well-known lifestyle diseases, diabetes and depression. The corresponding DIAs are shown in Table 2. The relationship between the diseases and the activities are shown in Figure 8.

We use three real-world activity datasets gathered for two different activity recognition studies [19,22]. Both of these studies have considered a home environment in which a set of sensors are tagged with daily life objects. The sensors were installed in everyday objects such as drawers, refrigerators, and containers to record activation/deactivation events (opening/closing events) as the subject carried out everyday activities. Kasteren *et al.* [22] have chosen an apartment of a 26-year-old man, deployed 14 sensors, and attached these to doors, cupboards, a refrigerator, and a toilet flush. They have collected data for 28 days. We refer this dataset as Subject 1. Tapia *et al.* [19] have used a similar setting. Tapia *et al.* [19] have collected data in two different single-person apartments. They have used 77 and 88 sensors respectively. They have collected data for 14 days in each apartment. We refer to these datasets as Subject 2 and Subject 3.

From these datasets, we have selected the following activities, “going out”, “toileting”, “showering”, “sleeping” and “eating” as a set of DIAs. We have also measured a DIA, “Movement”, from the distance traveled by a subject in a house. Additionally, the “weight” and “lighting” values are artificially generated from the average weight in the United States [31] and the Illuminating Engineering Society (IES) recommended indoor luminance [32] respectively. For example, for the Subject 1, weight and lighting values are set 189.8 pounds and 300 lux respectively based on the average weight of an adult male and the recommended indoor luminance.

We have used Leave-one-out cross-validation for measuring the activity recognition accuracy as well as for the disease prediction. Cross-validation permits such measurement even on small datasets [19]. We have used the window size of 60 s (1 min) for activity recognition since it is sufficiently long to be discriminative and short enough to provide high accuracy [22]. Additionally, we have used 600 s (10 min) to see the variation of accuracies.

### 4.2. Experimental Results

#### 4.2.1. Activity Classification

The purpose of this experiment is to see how accurate the activity classifier is in classifying the activities. For classification algorithm, we have adopted J48 decision tree in Weka [33].

**Figure 9.**
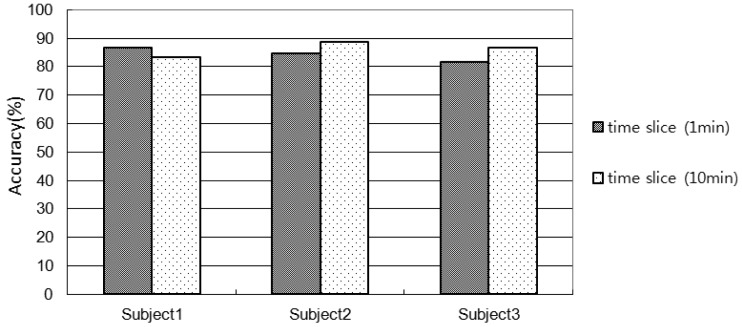
Activity recognition results.

Figure 9 shows the accuracy of activity recognition. The accuracies with 1min-window for Subject 1, 2, and 3 are 86.5%, 84.6%, 81.4%, respectively. The accuracies with 10 min-window for Subject 1, 2, and 3 are 83.4%, 88.6%, 86.4%, respectively.

It is observed that the lower the number of sensors used in an environment the shorter the window size is required. For example, the accuracy for Subject 1 of 10 min window size is lower than that of 1 min window size since they have used 14 sensors. On the other hand the accuracy for Subject 2 and 3 of 10 min windows size is higher than the 1 min window size since they have used more than 70 sensors. In comparison with Subject 2 and 3, in the Subject 1's environment only the key objects (frequently used for an activity) were embedded with sensors. If the window size is small, the chances of using less number of sensors (usually key sensors) are high and therefore the classifier would make less confusion. However, the scenario is opposite for the large window size. Even though, the activity recognition accuracies are high, however, it would be possible to improve the accuracy by introducing another robust classifier such as SVM.

#### 4.2.2. Lifestyle Diseases Prediction

**Figure 10.**
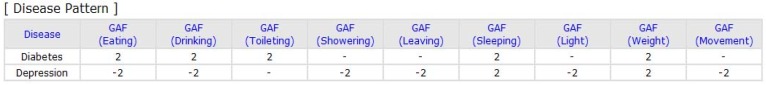
Disease patterns for diabetes and depression.

The purpose of this experiment is to evaluate how the framework would work for disease prediction. The disease prediction module takes the disease pattern, *DAP_disease_* and the daily activity pattern, *DAP_d_* as input and produces a probability of a lifestyle disease, *R_disease_*(*DAP_disease_, DAP_d_*) as the output. The disease pattern should be set by a doctor based on his expertise. Figure 10 shows the settings of disease patterns for the experiments.

**Figure 11.**
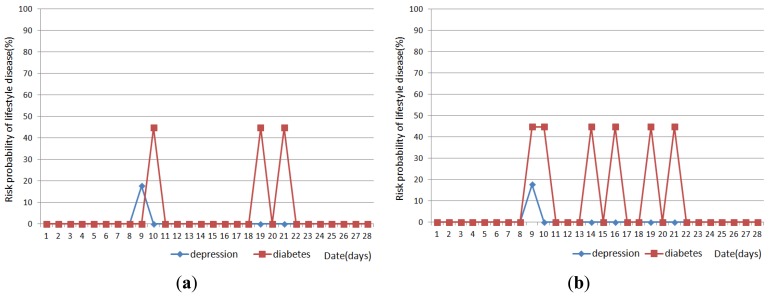
Results of lifestyle disease prediction for Subject 1. (**a**) Activity recognition with 1 min window; and (**b**) Activity recognition with 10 min window.

**Figure 12.**
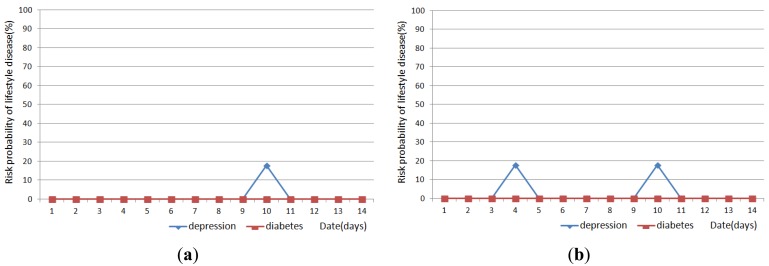
Results of lifestyle disease prediction for Subject 2. (**a**) Activity recognition with 1 min window; and (**b**) Activity recognition with 10 min window.

**Figure 13.**
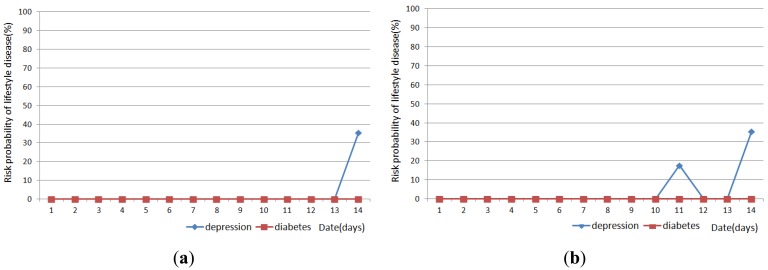
Results of lifestyle disease prediction for Subject 3. (**a**) Activity recognition with 1 min window; and (**b**) Activity recognition with 10 min window.

Figures 11, 12 and 13 show the result of disease prediction for Subject 1, Subject 2, and Subject 3 respectively. As we can see in the figures, the proposed method has the ability to determine the probability of lifestyle disease.

For example, the risk probabilities of diabetes and depression for Subject 2 and Subject 3 are 0% for most of the days, since the graded activity frequencies for diabetes and depression are within the range of regular activity frequency (RAF). However, the risk probabilities of diabetes and depression for Subject 1 are over than 0% for several days. In Figure 11(a), Subject 1 has 17.6% probability of depression on day 9. This means Subject 1 has irregular activity patterns that may lead to depression. Subject 1 had less movement during this day. The GAF for movement of day 9 was −1. This irregular pattern corresponds to initial signs and symptoms for depression such as “loss of interest in daily activities”. Subject 1 also has 44.7% probabilities of diabetes on day 10, 19 and 21. The GAF for toileting of day 10 is 2 and the GAFs for eating of day 19 and 21 were 2. These irregular patterns correspond to initial signs and symptoms for diabetes such as “increased urination” and “increased hunger.” Similar phenomenon can be observed in Figure 12 and 13. If the irregularities continue for a long period of time, a doctor can examine the possibility of the disease and suggests regular lifestyle.

Activity recognition result influences lifestyle disease prediction result. As shown in Figures 11, 12 and 13, the lifestyle disease prediction sometimes differs based on the window sizes used for activity recognition. This is because the incorrect activity recognition could lead to wrong GAF and DAP calculation. For example, in Figure 12, Subject 2 has 0% and 17.6% probabilities of depression on day 4 depending on window size. Since activity classifier with 10min window misrecognized “eating” to “idle”, GAF for eating is generated as −1. Subject 1 has more difference between 1 min and 10 min window sizes compared with Subjects 2 and 3. This is also due to the number of sensors used in the environment (as discussed in the previous section).

### 4.3. Discussion

The activity recognition is the most important module of the framework. The accuracy of this module is the key for the performance of disease prediction. If an activity is misrecognized, the framework generates incorrect GAFs and DAPs which would lead to wrong disease prediction. Therefore, by introducing a more robust activity recognition module, it would be possible to increase the accuracy of prediction.

The current version of the system works in a single user environment. However, by simply replacing the activity recognition module, it is possible to extend the system such that it works in a multi-user environment. Several algorithms [34–36] have already been proposed for activity recognition in a multi-user environment. We would be able to customize such an algorithm to fit in this framework.

## 5. Conclusions and Future Work

Long-term activity monitoring of a person could be helpful for managing lifestyle associated diseases. In this paper, we have proposed a framework for supervising lifestyle diseases using long-term activity monitoring. The framework is applicable to a home environment in which a set of sensors are embedded with the daily-life objects such that it is possible to determine the state of an object at any given time. This framework is hierarchical and comprises of three modules: activity recognition, activity pattern modeling and disease prediction.

The activity recognition module recognizes a user's activity from the set of objects used in a period of time. The activity pattern generation module generates the activity pattern per day from the user's activities. The disease prediction module evaluates the probability of a disease using the pattern generated by the activity generation module. We have shown that it is possible to estimate the likelihood of lifestyle diseases from the sensor data. We have also shown the viability of the proposed framework.

The current version of the framework uses the frequency of an activity as the primary source for disease inference. However, there could be other sources of irregularities, for example, the way of doing an activity, and the sequence of activities. In the next version of the framework we would explore such irregularities as the potential sources for lifestyle prediction.

The current version of the system does not work in a multi-user environment. Therefore, in the future version of the system we will be exploring different multi-user based activity recognition algorithms such that the system can be extended for multi-user scenarios.

## Figures and Tables

**Table 1.** Example of DIAs of Diabetes and Depression.

**Lifestyle Disease**	**Initial Signs and Symptoms**	**DIAs**
Depression	Down feeling	Activity in mild illumination
	Loss of interest in daily activities	infrequent traversal or leaving house, unhygienic activity, less talking,
	Sleep changes (oversleeping is less common)	Sleeping disorder
	Appetite or weight changes	less eating weight loss

Diabetes	increased thirst	frequent drinking
	increased hunger	frequent eating
	fatigue	frequent sleeping
	increased urination	frequent toileting
	unintended weight loss	low weight

**Table 2.** The lifestyle diseases and the corresponding DIAs.

**Lifestyle Disease**	**DIAs**
Depression	Activity in mild illumination
	infrequent traversal or leaving house, unhygienic activity, less talking,
	Sleeping disorder
	less eating, weight loss

Diabetes	frequent drinking
	frequent eating
	frequent sleeping
	frequent toileting
	low weight
